# Usefulness of Ultrasonography for Diagnosis of Small Bowel Tumors: A Comparison Between Ultrasonography and Endoscopic Modalities

**DOI:** 10.1097/MD.0000000000001464

**Published:** 2015-10-09

**Authors:** Minoru Fujita, Noriaki Manabe, Keisuke Honda, Takahisa Murao, Motoyasu Osawa, Ryosuke Kawai, Takashi Akiyama, Akiko Shiotani, Ken Haruma, Jiro Hata

**Affiliations:** From the Division of Gastroenterology, Department of Internal Medicine (MF, TM, MO, AS); Division of Endoscopy and Ultrasound, Department of Clinical Pathology and Laboratory Medicine (NM, RK, JH); Department of General Medicine (KH); Department of Pathology (TA); Department of General Internal Medicine, Kawasaki Medical School, Kurashiki, Japan (KH); and Department of Clinical Nutrition, Faculty of Health Science and Technology, Kawasaki University of Medical Welfare, Kurashiki, Japan (KH).

## Abstract

Ultrasonography is a standard, noninvasive modality used to evaluate patients with gastrointestinal diseases. This study assessed the usefulness of ultrasonography in the detection of small bowel tumors.

This study enrolled 558 consecutive patients (295 males, 263 females; mean age 71.1 years) who underwent ultrasonography before capsule endoscopy and/or balloon-assisted endoscopy. Ultrasonographic detection of small bowel tumors was compared with detection by capsule endoscopy and/or balloon-assisted endoscopy. In addition, factors affecting small bowel tumor detection by ultrasonography and clinical characteristics of patients with small bowel tumors undetected by ultrasonography were evaluated.

Ninety-seven tumors (52 benign, 45 malignant) detected by capsule endoscopy and/or balloon-assisted endoscopy were retrospectively analyzed. The sensitivity and specificity of ultrasonography in the detection of small bowel tumors were 50.5% (47/93) and 100% (465/465), respectively. If we restricted patients to those with a tumor >20 mm in size, its detection ratio would become higher (91.7%): the ratio of submucosal tumor >20 mm in size was 85.7% (6/7) and that of partial and circumferential ulcerative tumors >20 mm in size was 96.9% (31/32), respectively. Small bowel tumors detected by ultrasonography (mean 33.2 mm) were significantly larger than those undetected by ultrasonography (mean 8.7 mm). The percentage of small bowel tumors located in the ileum detected by ultrasonography (70.6%) was significantly higher than those undetected by ultrasonography (29.4%). Of the 46 small bowel tumors undetected by ultrasonography, 42 (91.3%) were benign tumors with good clinical prognosis.

Ultrasonography is a useful modality for detecting larger small bowel tumors and ulcerative lesions. Ultrasonography should be considered a first-line modality for patients suspected of having small bowel tumors, because most small bowel tumors undetected by ultrasonography were benign tumors with good clinical prognosis.

## INTRODUCTION

The small bowel is the longest part of the digestive tract, accounting for 75% of the total length and 90% of the mucosal surface of the alimentary tract. The small bowel is not a common site for tumor development, inasmuch as only 3–6% of all gastrointestinal (GI) neoplasms and 1–3% of all primary GI malignancies occur in this organ.^1–3^ A recent epidemiological study, however, found that the overall incidence of malignant small bowel tumors (SBTs) has increased markedly in the United States.^4^ The clinical diagnosis of SBTs has been difficult because of the lack of specific clinical symptoms and effective diagnostic approaches.^2,3,5,6^ Primary and secondary SBTs are sometimes incidentally diagnosed by obscure GI bleeding and/or acute abdominal pain.^6,8^

More recently, endoscopic methods, including capsule endoscopy (CE) and balloon-assisted endoscopy (BAE), have been developed to evaluate diseases of the small intestine. Although these methods have been shown to be useful in the diagnosis and treatment of small intestinal lesions,^7–11^ these procedures have several limitations. CE cannot be used in patients with small bowel strictures, nor can it be used to biopsy tissue samples,^11–13^ whereas BAE is an invasive and complex procedure, with the examination requiring substantial time to complete.^14^

Ultrasonography (US) is one of the most commonly used imaging modalities in patients with GI diseases, including inflammatory bowel diseases and GI obstruction.^15–19^ In contrast to other abdominal examination methods, US has several advantages, including being radiation-free, noninvasive, and cost-effective, as well as providing real-time scanning.^20,21^ To date, however, few studies have assessed its usefulness in the detection of SBTs.^22^ This study therefore compared the ability of US to detect SBTs with detection by CE and/or BAE, as well as evaluating the factors affecting SBT detection by US.

## PATIENTS AND METHODS

### Patients

This retrospective cross-sectional study of consecutive patients suspected of SBTs was performed at Kawasaki Medical School from January 2007 to March 2013. After routine US evaluation of parenchymal organs, including the liver, kidneys, pancreas, and spleen, all patients underwent US evaluation of the GI tract before CE and/or BAE. This study was approved by the local ethics committee, and authorization for the use of medical records for research purposes was confirmed before access to these records was obtained.

## METHODS

### Data From Medical Records

Patient characteristics investigated included age, sex, body mass index (BMI), symptoms at the time of initial diagnosis, US findings, CE findings, BAE findings, and final diagnosis, including pathological findings of resected specimens.

### US Procedure

Routine abdominal US was performed using the SSA-390A system (Toshiba Medical Systems Co, Ltd, Otawara, Japan), with 3.5-MHz curved and 7.5-MHz linear array transducers, as previously described.^11,22^ Two US operators, each with >10 years of experience, screened the GI tract. They were blinded to the results of other imaging procedures but had general information about patients’ physical findings and results of blood tests.

### CE Procedure

CE was performed using an M2A/PillCamTM SB, along with the Reporting and Processing of Images and Data (rapid) (2–5.5) application software program (Given Imaging, Yokneam, Israel), as described.^9–11^

### DBE Procedure

Double-balloon endoscopy (EN450P5, EN450T5; Fujifilm Inc, Tokyo, Japan), was performed using antegrade and/or retrograde approaches, as previously described.^7,8^ For retrograde BAE, bowel preparation was required, as for colonoscopy. Antegrade BAE was performed after an overnight fast. All BAE procedures were performed under conscious sedation.

### Study Protocol and Evaluation

This study evaluated three parameters: the detectability of SBTs by US compared with CE and/or BAE, factors affecting the detection rate of US, and clinical characteristics of patients with SBTs undetected by US. All patients underwent US before CE and/or BAE. If the lesions detected by US were large (>15 mm) or were accompanied by massive bleeding, BAE was performed first. All other patients underwent CE. The US images were evaluated for the presence and features of SBTs. CE or BAE was performed within 2 weeks of US examination. If CE or BAE detected multiple lesions, the largest was analyzed. The size, shape, and location of lesions detected by US were compared with the same factors on CE or BAE. Tumor size was evaluated as the major axis length, measured on surgically or endoscopically resected specimens, small bowel radiograms, or endoscopic images. Tumor shapes were classified endoscopically as granular lateral spreading tumors, polypoid lesions, submucosal lesions, circumscribed lesions, and circumferential ulcerative lesions,^22^ and tumor location as the jejunum and ileum. SBTs located in the epigastric and/or left hypochondrium were defined as being in the upper jejunum, those in the periumbilical and/or right lateral abdominal region as being in the lower jejunum, and those in the left lower abdominal region and/or pelvic cavity as being in the ileum. This study was approved by the research ethics committee of Kawasaki Medical School and Hospital (number 1299).

### Statistical Analysis

Values expressed as mean ± standard deviation (SD) were compared using Student's *t* tests. The chi square test was used to compare percentages and to assess the independence of two qualitative variables. In all analyses, *P* < 0.05 was considered to be significant. All statistical analyses were performed using GraphPad Prism 5 for Windows (GraphPad Software Inc, San Diego, CA).

## RESULTS

### Patients’ Characteristics

This study enrolled 558 consecutive patients, 295 males and 263 females, of mean ± SD age 70.1 ± 12.4 years. Of these patients, 373 (67.2%) showed symptoms related to obscure GI bleeding, 75 (13.4%) had abdominal pain, 46 (8.6%) had anemia, 41 (7.3%) underwent detailed examinations, 25 (4.4%) had abdominal obstruction suggesting bowel stenosis, and 44 (7.8%) had other symptoms. There were some overlaps among symptoms.

### Clinical Characteristics of SBTs Detected by CE and/or BAE

**FIGURE 1:**
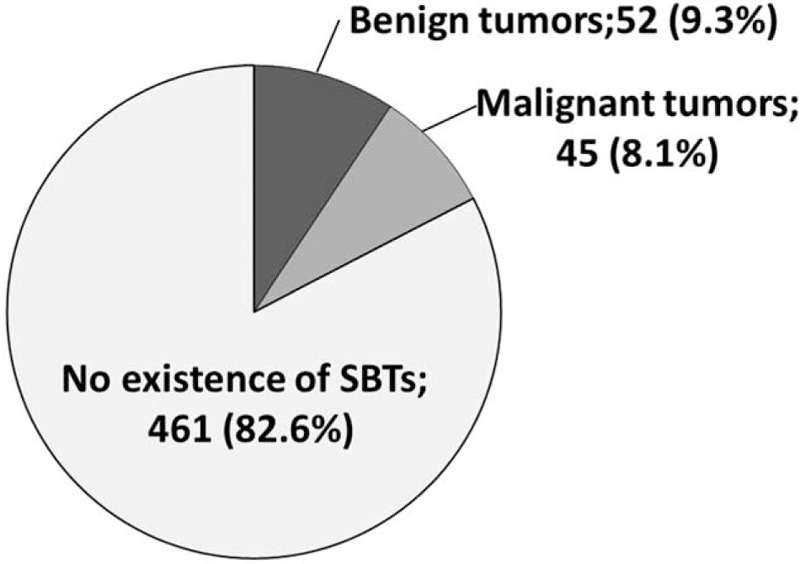
Numbers of SBTs detected by small bowel endoscopy. SBTs = . small bowel tumors.

SBTs were detected by CE and/or BAE in 93 (16.7%) patients (Figure 1), Benign SBTs were observed in 52 patients (9.3%, mean size; 16.9 ± 1.7 mm), including 24 (46.2%) with lymphangiomas; 6 (11.5%) with hamartomatous polyps; 2 (3.8%) with inflammatory fibroid polyps; 1 each (1.9%) with a lipoma, a pyogenic granuloma, and an adenoma; and 17 (32.7%) with other lesions. Malignant SBTs were detected in 45 patients (8.1%, mean size; 36.2 ± 0.4 mm), including 31 (68.9%) with malignant lymphomas, 6 (13.3%) with adenocarcinomas, 4 (8.9%) with gastrointestinal stromal tumors (GISTs), 3 (6.7%) with metastatic tumors, and 1 (2.2%) with a carcinoid tumor.

### Tumor Detectability on US Compared with CE and/or BAE

Table 1 shows the detectability of SBTs by US and by CE and/or BAE. US detected SBTs in 51 patients (9.1%), but failed to detect SBTs detected by CE and/or BAE in 46 patients (8.2%), whereas 461 patients (82.6%) were diagnosed as negative by both US and CE and/or BAE. The sensitivity, specificity, positive predictive value (PPV), and negative predictive value (NPV) of US in the detection of SBTs were 53.1%, 100%, 100%, and 90.9%, respectively.

**TABLE 1:**
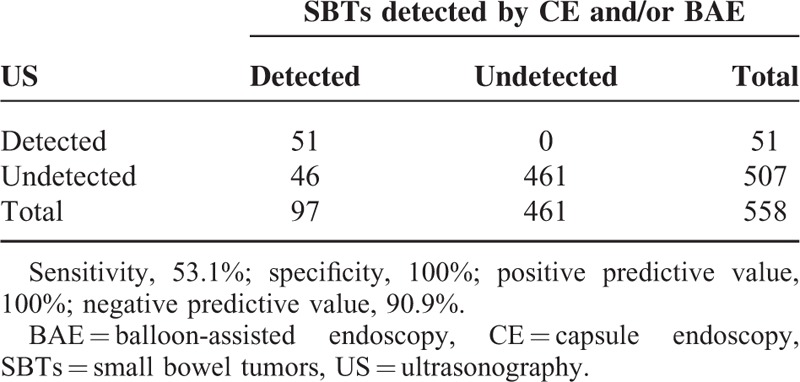
Detectability of SBTs by US Compared with CE and/or BAE

### US Detectability of SBTs by Shape and Size

**FIGURE 2:**
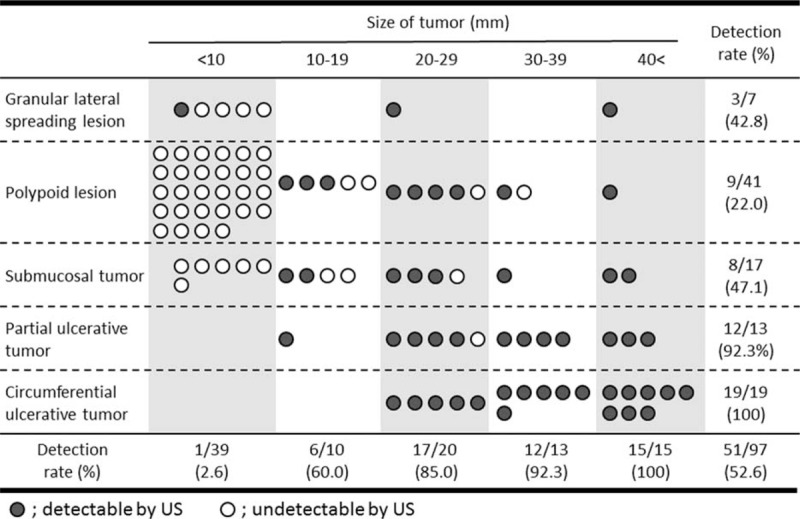
Rate of US detection of SBTs as a function of tumor shape and size. SBTs =  small bowel tumors, US = ultrasonography.

The ability of US to detect SBTs by their shape and size is shown in Figure 2. Only one (2.6%) of 39 tumors <10 mm was detected by US, compared with 44 (91.7%) of 48 tumors >20 mm. Of the 33 partial ulcerative and circumferential tumors, 32 (96.9%) could be detected by US, compared with only 1 (3.0%) of 33 polypoid lesions and granular lateral spreading lesions <10 mm in size. If we restricted patients to those with a tumor >20 mm in size, its detection ratio would become higher (91.7%): the ratio of submucosal tumor >20 mm in size was 85.7% (6/7) and that of partial and circumferential ulcerative tumors >20 mm in size was 96.9% (31/32), respectively.

### Factors Affecting US Detection Rate

**FIGURE 3:**
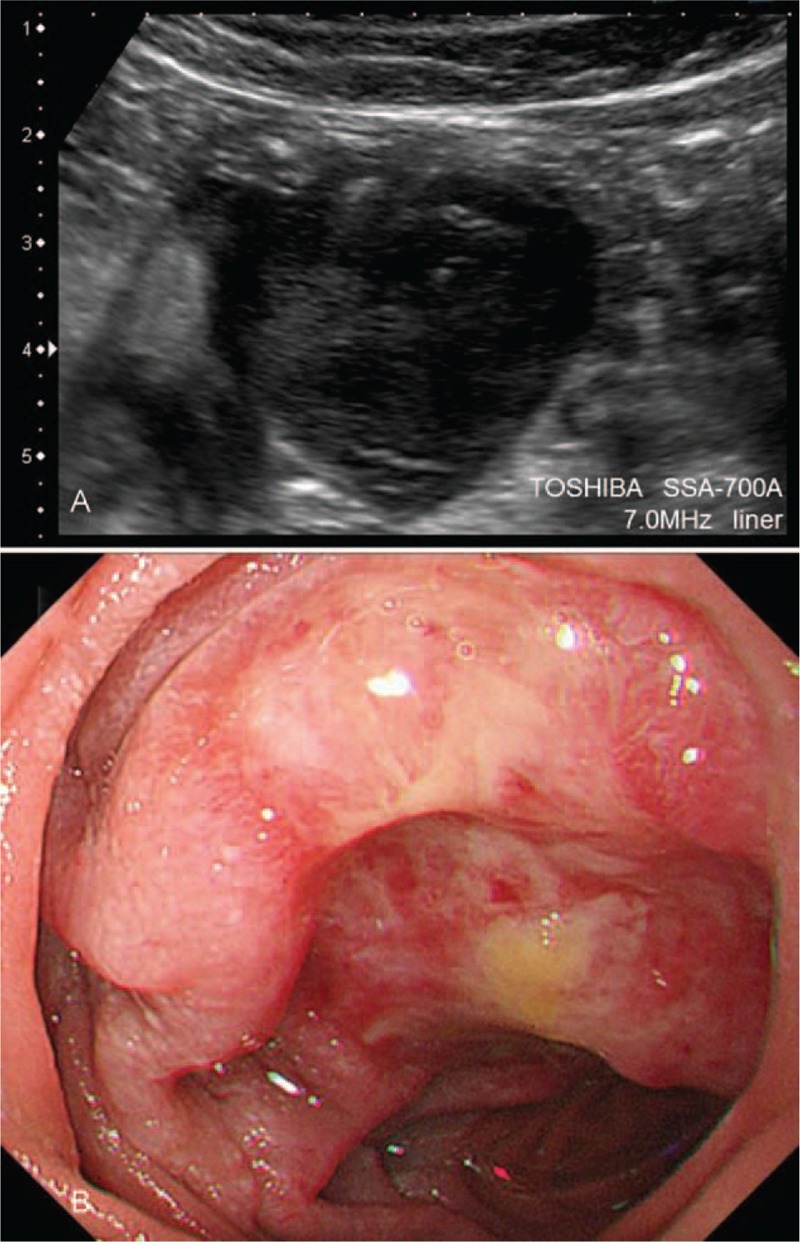
Results in a patient with SBT of the jejunum detected by both US and BAE. Tumor size was 30 × 22 mm, and the histological diagnosis was diffuse large B cell lymphoma. (A) US results, showing a very low echoic round mass lesion in the periumbilical region. (B) BAE results, confirming the presence of a partial ulcerative mass in the distal jejunum. BAE = balloon-assisted endoscopy, SBTs =  small bowel tumors, US = ultrasonography.

**FIGURE 4:**
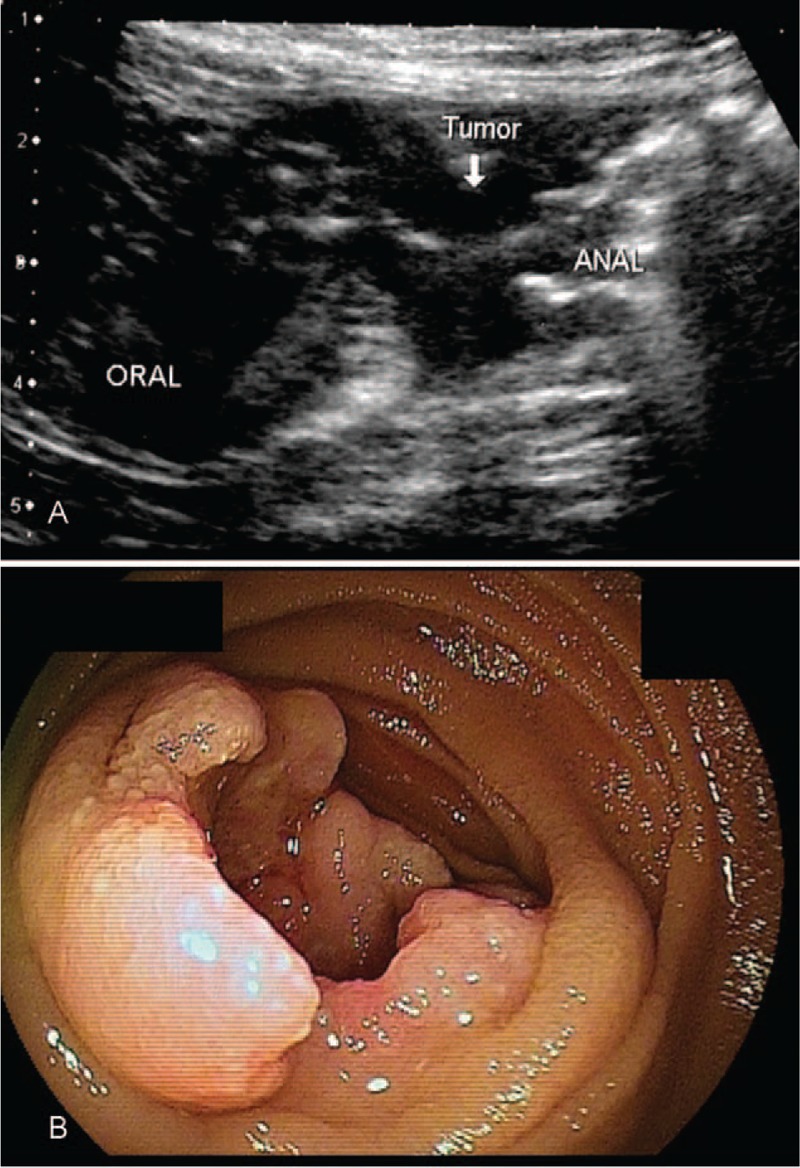
Results in a patient with SBT of the jejunum detected by both US and BAE. Tumor size was 15 × 13 mm, and the histological diagnosis was jejunal cancer. (A) US results, showing a low-echoic mass surrounding the entire circumference of the small intestinal wall in the epigastric region. (B) BAE results, confirming the presence of a circumferential ulcerative mass in the upper jejunum. BAE = balloon-assisted endoscopy, SBTs =  small bowel tumors, US = ultrasonography.

**FIGURE 5:**
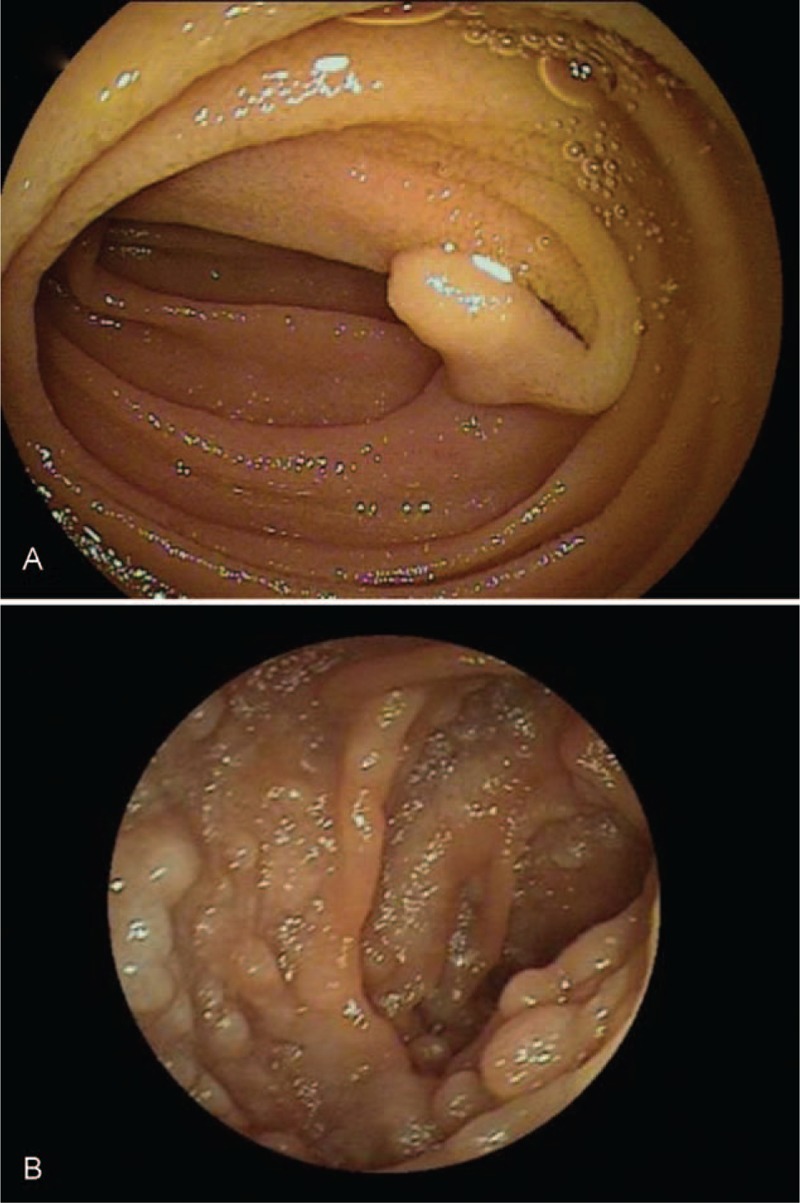
Endoscopic images of SBTs undetected by US. Few polypoid lesions or superficial abnormalities were detected by US. (A) Small polypoid lesion in the jejunum. (B) Follicular lymphoma in the upper jejunum. SBTs =  small bowel tumors, US = ultrasonography.

Table 2 shows the factors affecting US detection rate among patients with SBTs. Tumors detected by US were significantly larger than those undetected by US (33.2 ± 16.3 mm vs 8.7 ± 8.6 mm, *P* = 0.00015). Tumor location also differed significantly in these 2 groups (*P* = 0.014), with localization in the ileum being significantly more common for SBTs detected than undetected by US. The sex, age, and BMI (*P* = 0.56) of patients with tumors detected and undetected by US did not differ significantly. Typical SBTs detected by US are shown in Figures 3 and 4, and typical SBTs undetected by US are shown in Figure 5.

**TABLE 2:**
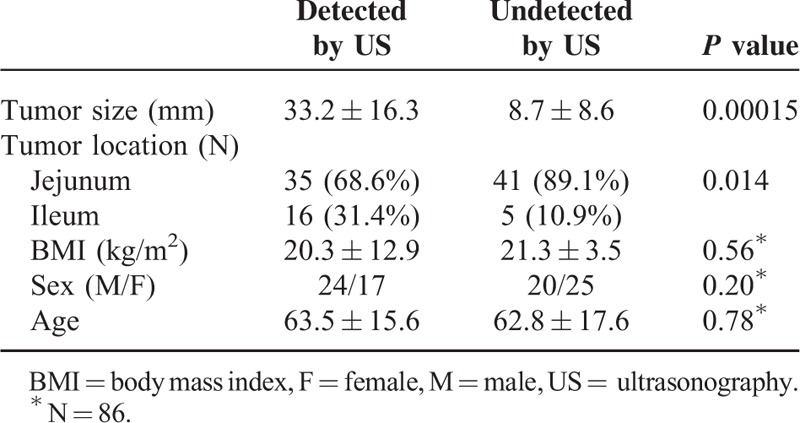
Factors Affecting US Detection Rate

### Clinical Characteristics of Patients with SBTs Undetected by US

**FIGURE 6:**
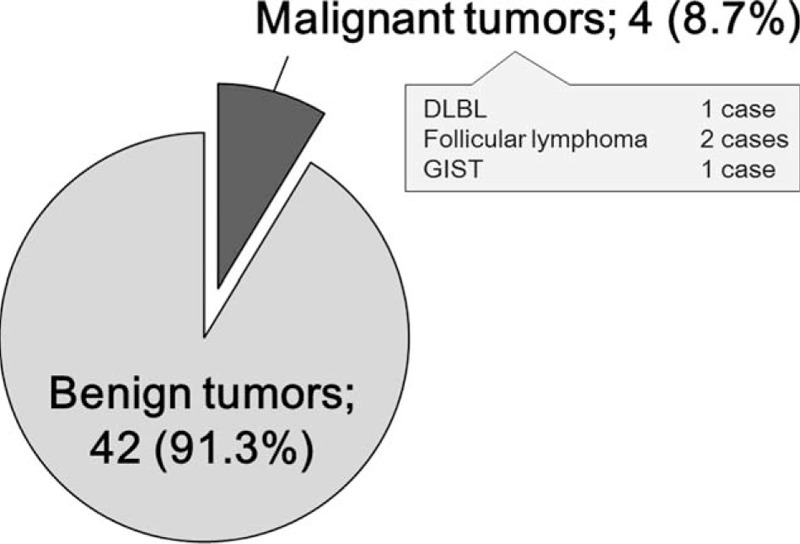
Descriptions of patients with SBTs undetected by US examination. SBTs =  small bowel tumors, US = ultrasonography.

Of the 52 patients with SBTs undetected by US, 42 (80.8%) had benign tumors (Figure 6). In contrast, of the 45 patients with malignant tumors, only 4 (8.9%) were undetected by US, including 2 patients with follicular lymphoma and 1 each with diffuse large B cell lymphoma and GIST. Three of these tumors were located in the duodenum and upper jejunum and 2 patients had small, multiple lesions (Table 3).

**TABLE 3:**
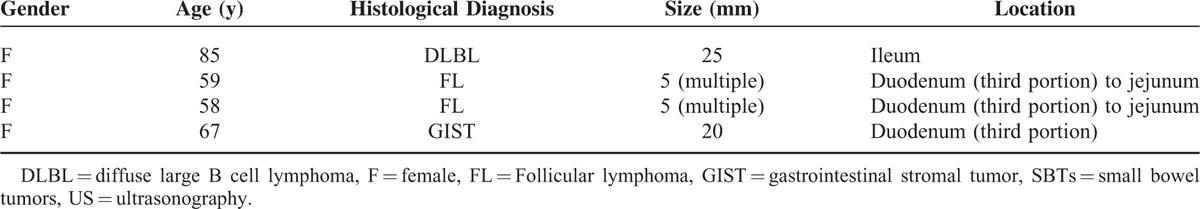
Descriptions of Individual Patients with Malignant SBTs Undetected by US

## DISCUSSION

This study compared the detectability of SBTs by US with detectability by CE and/or BAE, finding that US had a sensitivity of 53.1% and a specificity of 100%. The US detection rate of SBTs >20 mm in longest diameter was 91.7% (44/48). Moreover, >90% of SBTs undetected by US were benign tumors, which have been generally regarded as having a good clinical course. These findings suggested that US examination may be a useful first-line modality for patients suspected of having SBTs.

The sensitivity, specificity, PPV, and NPV rates of US in the detection of SBTs were found to be 53.1%, 100%, 100%, and 90.9%, respectively. In comparison, 2 previous studies reported sensitivity rates of US 26.4% and 72.0%.^22,23^ These discrepancies may have been due to differences in patient populations, reference diagnostic modalities, or experience of the US operator. In this study, each US operator had >10 years of experience. US examination of the GI tract in our institution has become routine, with all operators having >10 years of experience.^15,16,21,24^ Moreover, many US operators in other hospitals throughout Japan have >10 years of experience.

This study found that the overall rate of detection of SBTs >20 mm on US was 91.7% (44/48). Moreover, US successfully detected all circumferential tumors and 12 of 13 (92.3%) partial ulcerative tumors, higher rates than previously observed.^22^ The severe obstruction associated with large-sized SBTs is a risk factor for capsule retention. Moreover, patients with a severe condition may be contraindicated for BAE because of its invasiveness. US examination may be an alternative, because of its noninvasiveness. In contrast, the US detection rate of SBTs <20 mm was only 14.3% (7/49), suggesting that US examination may be unsuitable for small-sized SBTs, although such patients could undergo CE and/or BAE.

The diagnostic quality of US examinations depends on several factors, including the resolution of the equipment, the experience of the operator, artifacts produced by intestinal gas, and the patient's body habitus, including BMI and the thickness of the fatty layer of the anterior abdominal wall.^25–31^ Unexpectedly, BMI was not associated with the rate of SBT detection by US. Because of the retrospective nature of this study, the thickness of the fatty layer of the anterior abdominal wall could not be investigated. The enrolled patients tended to have low BMI, which may explain the lack of difference between the 2 groups. However, other studies have reported that BMI was unrelated to SBT detectability by US.^32,33^

US and computed tomography (CT) showed similar rates of detection of acute appendicitis and colonic diverticulitis (CT).^34,35^ In this study, ileocecal SBTs had a higher rate of detection than lesions in other locations. Anatomically, ileocecal lesions are located more superficially in the abdominal cavity, making these SBTs easier to detect by US than lesions in other locations.

SBTs detected by US were significantly larger than those undetected by US in this study, providing further evidence that small-sized SBTs are difficult to detect by US. Our study showed that US was able to detect only 2.6% of SBTs <10 mm in size, similar to a study reporting that US was unable to detect any SBTs <10 mm in size.^22^ US was also unable to detect 4 SBTs >30 mm, 3 of which were located in the duodenum and/or upper jejunum and 1 on the deep side of the pelvic cavity, suggesting that the latter may be difficult to detect by US. However, >90% of SBTs undetected by US were benign tumors with good clinical prognosis. Further studies are necessary to confirm this finding.

This study had several limitations. First, the detection rate of SBTs in asymptomatic patients has been unclear, although we have had experience detecting SBTs incidentally during routine US examination. However, it is difficult to determine this rate, because many asymptomatic patients are not evaluated. Second, the correlation between US operator experience and the rate of SBT detection is unclear, although several studies have shown that operator experience was associated with the rate of detection of GI diseases.^36–38^ Third, this study excluded patients unable to undergo CE and/or BAE after US examination because of severe general condition. Inclusion of these patients may have increased the sensitivity and specificity of US. Fourth, SBT detectability by US examination was not compared with detectability by CT and/or magnetic resonance imaging (MRI). Several reports have evaluated the advantages and disadvantages of CT and MRI for the diagnosis of SBTs.^2,39,40^ Finally, the number of included patients was relatively small, indicating the need for studies in larger numbers of patients with SBTs.

## SUMMARY

CE and BAE are considered useful diagnostic modalities in patients suspected of having SBTs. However, these examinations have several disadvantages, including their cost and invasiveness. Moreover, they may be impossible to perform in patients with poor performance status and/or severe bowel obstructions. In contrast, US is cost effective, noninvasive, and easy to use^29^ and can performed in all patients. This study showed that 91.1% (41/45) of malignant SBTs could be detected by US and that most SBTs undetected by US were benign tumors with good clinical prognosis. US examination may be a useful first-line diagnostic modality in patients suspected of SBTs. Additional studies in larger numbers of patients are needed to confirm these findings.
